# Formation Mechanism of a Nano‐Ring of Bismuth Cations and Mono‐Lacunary Keggin‐Type Phosphomolybdate

**DOI:** 10.1002/chem.202200079

**Published:** 2022-04-01

**Authors:** Inês C. B. Martins, Dominik Al‐Sabbagh, Ursula Bentrup, Julien Marquardt, Thomas Schmid, Ernesto Scoppola, Werner Kraus, Tomasz M. Stawski, Ana Guilherme Buzanich, Kirill V. Yusenko, Steffen Weidner, Franziska Emmerling

**Affiliations:** ^1^ BAM Federal Institute for Materials Research and Testing Richard-Willstätter-Str.11 12489 Berlin Germany; ^2^ Department of Chemistry Humboldt-Universität zu Berlin Brook-Taylor-Str. 2 12489 Berlin Germany; ^3^ Leibniz-Institut für Katalyse e. V. (LIKAT) Albert-Einstein-Str. 29a 18059 Rostock Germany; ^4^ School of Analytical Sciences Adlershof (SALSA) Humboldt-Universität zu Berlin Unter den Linden 6 10099 Berlin Germany; ^5^ Biomaterials, Hierarchical Structure of Biological and Bio-inspired Materials Max Planck Institute of Colloids and Interfaces Am Mühlenberg 1 14476 Potsdam Germany

**Keywords:** bismuth, in situ EXAFS, in situ SAXS/WAXS, lacunary Keggin ion, polyoxometalates, self-assembly

## Abstract

A new hetero‐bimetallic polyoxometalate (POM) nano‐ring was synthesized in a one‐pot procedure. The structure consists of tetrameric units containing four bismuth‐substituted monolacunary Keggin anions including distorted [BiO_8_] cubes. The nano‐ring is formed via self‐assembly from metal precursors in aqueous acidic medium. The compound (NH_4_)_16_[(BiPMo_11_O_39_)_4_] ⋅ 22 H_2_O; **(P_4_Bi_4_Mo_44_)** was characterized by single‐crystal X‐ray diffraction, extended X‐ray absorption fine structure spectroscopy (EXAFS), Raman spectroscopy, matrix‐assisted laser desorption/ionisation‐time of flight mass spectrometry (MALDI‐TOF), and thermogravimetry/differential scanning calorimetry mass spectrometry (TG‐DSC‐MS). The formation of the nano‐ring in solution was studied by time‐resolved in situ small‐ and wide‐angle X‐ray scattering (SAXS/WAXS) and in situ EXAFS measurements at the Mo−K and the Bi−L_3_ edge indicating a two‐step process consisting of condensation of Mo‐anions and formation of Bi−Mo‐units followed by a rapid self‐assembly to yield the final tetrameric ring structure.

## Graphical abstract

**Formation of a** tetrameric heteropolyoxometalate ring of bismuth‐containing lacunary phosphomolybdate clusters.

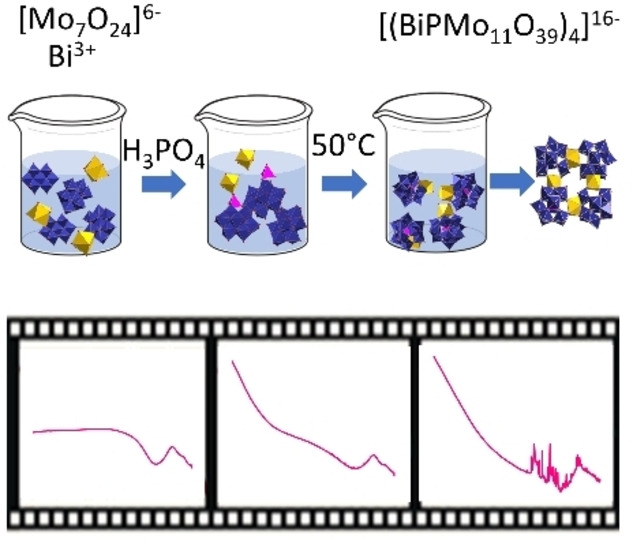

Polyoxometalates (POMs) are structurally well‐defined nanosized metal‐oxo cluster anions consisting of early transition metals like niobium, vanadium, molybdenum, or tungsten cations in high oxidation states.^[1]^ The structural variety of POMs offers the possibility to fine‐tune their properties towards applications in biomedicine,^[2]^ catalysis,^[3]^ and material chemistry.^[4]^ Among the POM structures derivatives of the Keggin‐type^[5]^ are especially interesting offering access to different substitutions within the POM‐based materials. The variability is particularly well‐established in polyoxomolybdate and ‐oxotungstate chemistry, where clusters featuring one or several metal cation binding sites (i. e., lacunary POMs) are formed.^[6]^ Particularly, trivalent cations, such as Ce^3+^, Gd^3+^, and Co^3+^ have been employed, since their high coordination number and flexible environment, allows for the formation of more complex architectures and superstructures.^[7]^

Among the main group elements, Bi^3+^ is used for connecting POM structures.[^2a^, ^2b^, ^7d^, ^8^] Bi^3+^‐containing POMs are investigated for their use in biomedical applications,^[2b]^ as proton conductors,[^7d^, ^9^] and as catalysts.^[10]^ In Bi‐containing POMs, Bi^3+^ cations typically adopt a trigonal‐pyramidal coordination geometry.[^8a^, ^10^, ^11^] Hanaya et al. reported the successful preparation of three types of bismuth‐tungsten oxide nanoclusters using lacunary silico‐tungstates. Structure‐directing properties of Bi^3+^ lone electron pair result in the formation of binuclear and tetranuclear cluster architectures.^[8a]^

Villanneau et al. reported two bismuth‐substituted structures [Bi{M_5_O_13_(OMe)_4_(NO)}_2_]^3−^ (M=Mo, W), in which the Bi^3+^ ions connect two monolacunary Lindqvist‐type clusters, resulting in an unusual 8‐fold coordination geometry around Bi^3+^.^[12]^ First heteropolytungstates exhibiting a similar 8‐fold square‐antiprismatic coordination geometry were characterized recently by Kortz et al.^[8b]^ and Sadakane and coworkers.^[13]^ Here, the authors used the structurally well‐defined coordination sites of lacunary POMs, allowing the construction of bismuth oxide clusters. Liu et al. reported a molecular cerium‐bismuth tungstate superstructure where Bi^3+^ ions incorporated into tungstate clusters prevented full closure of tungstate shells.^[7d]^ Bismuth ions are also used as capping group to stabilize highly anionic POMs.^[14]^

Despite the increasing interest in bismuth substituted POMs, little is known about the mechanism of formation of bismuth‐functionalized polyoxoanions and how the Bi‐coordination environment can be tailored.

Herein, we report the synthesis of an unprecedented ring structure based on lacunary polyoxomolybdate ions and bismuth cations in acid aqueous solution. The formation mechanism was investigated using time‐resolved in situ (TRIS) extended X‐ray absorption spectroscopy (EXAFS) and in situ small‐ and wide‐angle X‐ray scattering (SAXS/WAXS). The data suggest a two‐step formation process and a crucial influence of the temperature regime. The new ring structure was fully characterized using different solid‐state analytical methods such as X‐ray diffraction, Raman spectroscopy, MALDI‐TOF mass spectrometry, elemental and thermal analysis.

The heteropolyoxometalate (NH_4_)_16_[Bi_4_P_4_Mo_44_O_156_] ⋅ 22 H_2_O **(P_4_Bi_4_Mo_44_)** (see Figure 1) was obtained by mixing solutions of ammonium heptamolybdate (AHM), bismuth nitrate, and phosphoric acid (H_3_PO_4_) at elevated temperature (50 °C). During addition of the bismuth nitrate solution to an AHM solution, a white precipitate was immediately formed. After addition of phosphoric acid, the colour of the slurry changed gradually from bright yellow to orange. The precipitate was subsequently filtered and washed in water. From this solution suitable crystals were derived upon evaporation of the solvent in air.

**Figure 1:**
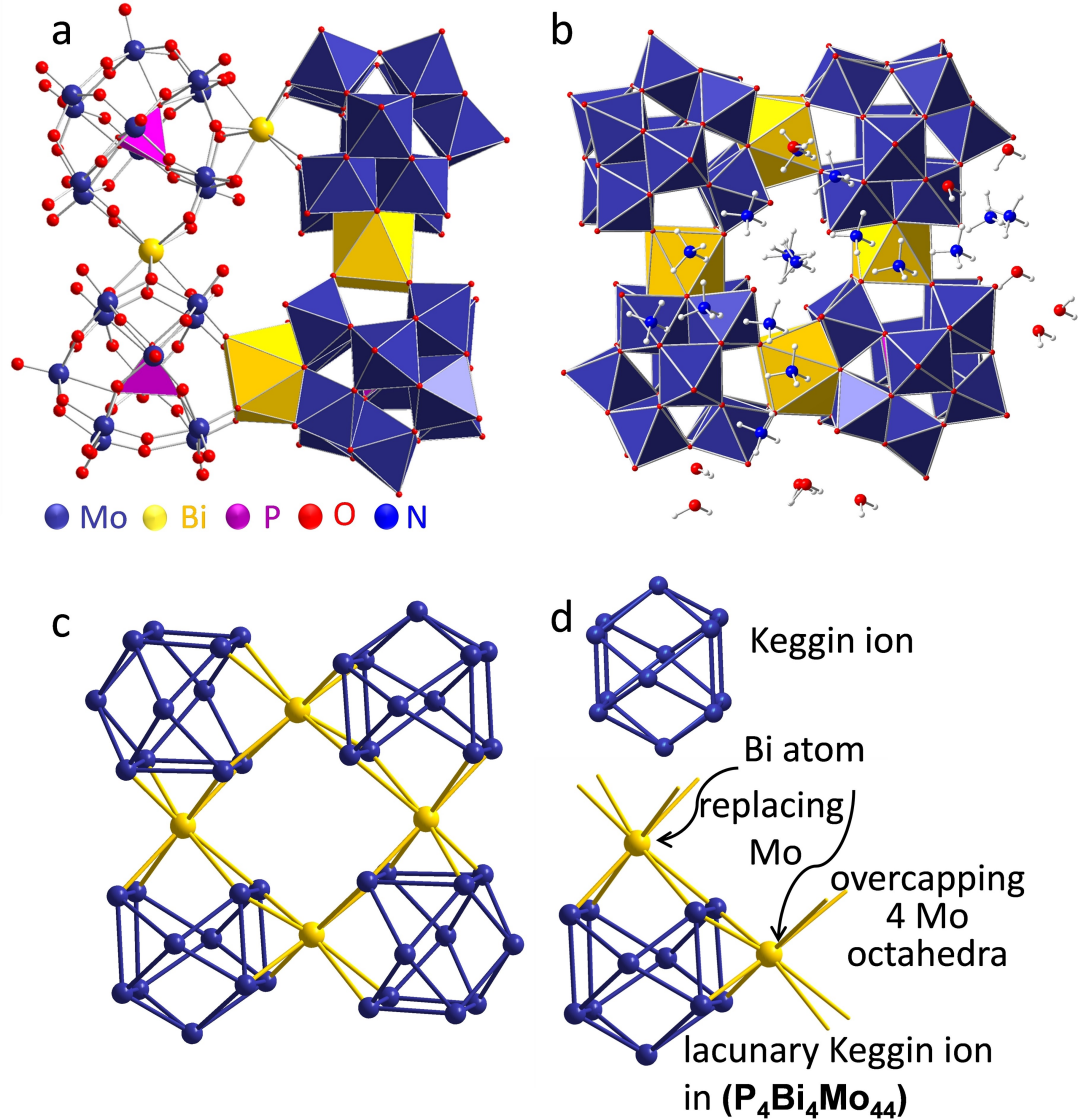
Representation of the nano‐ring structure in (P_4_Bi_4_Mo_44_) as identified by single‐crystal X‐ray diffraction. a) The polyhedra around Bi (yellow), Mo (blue), and around P (violet) are shown in the right half of the molecule. b) Polyhedron representation including water molecules and ammonium ions. c) Structure representation highlighting the metal framework in the nano‐ring, and d) the relation of the lacunary ion to the parental Keggin structure. The bonds drawn among metal atoms are an indication of the secondary building units and not covalent bonds.

The X‐ray diffraction patterns (see Figure S2 in Supporting Information) of the product are in good agreement with the simulated powder pattern calculated from the crystal structure.

The crystal structure of the heteropolyoxometalate **1** is assembled by four monolacunary Keggin anions [PMo_11_O_39_]^7−^ connected via four Bi^3+^ cations. The bismuth atoms substitute one Mo position in an ideal α‐Keggin anion ([α‐PMo_11_O_39_Bi]^4−^) and connect to four MoO_6_ octahedra of an adjacent Keggin ion. Each Bi^3+^ is coordinated in an 8‐fold coordination with square‐antiprismatic geometry. The resulting ring structure adopts a *C*_2*h*_ symmetry and consists of a ring with a size of 2.3×2.3×1.4 nm^3^. This ring structure is surrounded by water and ammonium molecules (Figure 1b).

Raman investigations were performed to elucidate the crucial steps during the formation (see Figure 2). The Raman spectra were measured from liquid samples of the ongoing synthesis by extracting aliquots of the reaction solution after each synthesis step, which were placed under a Raman microscope.^[15]^

**Figure 2:**
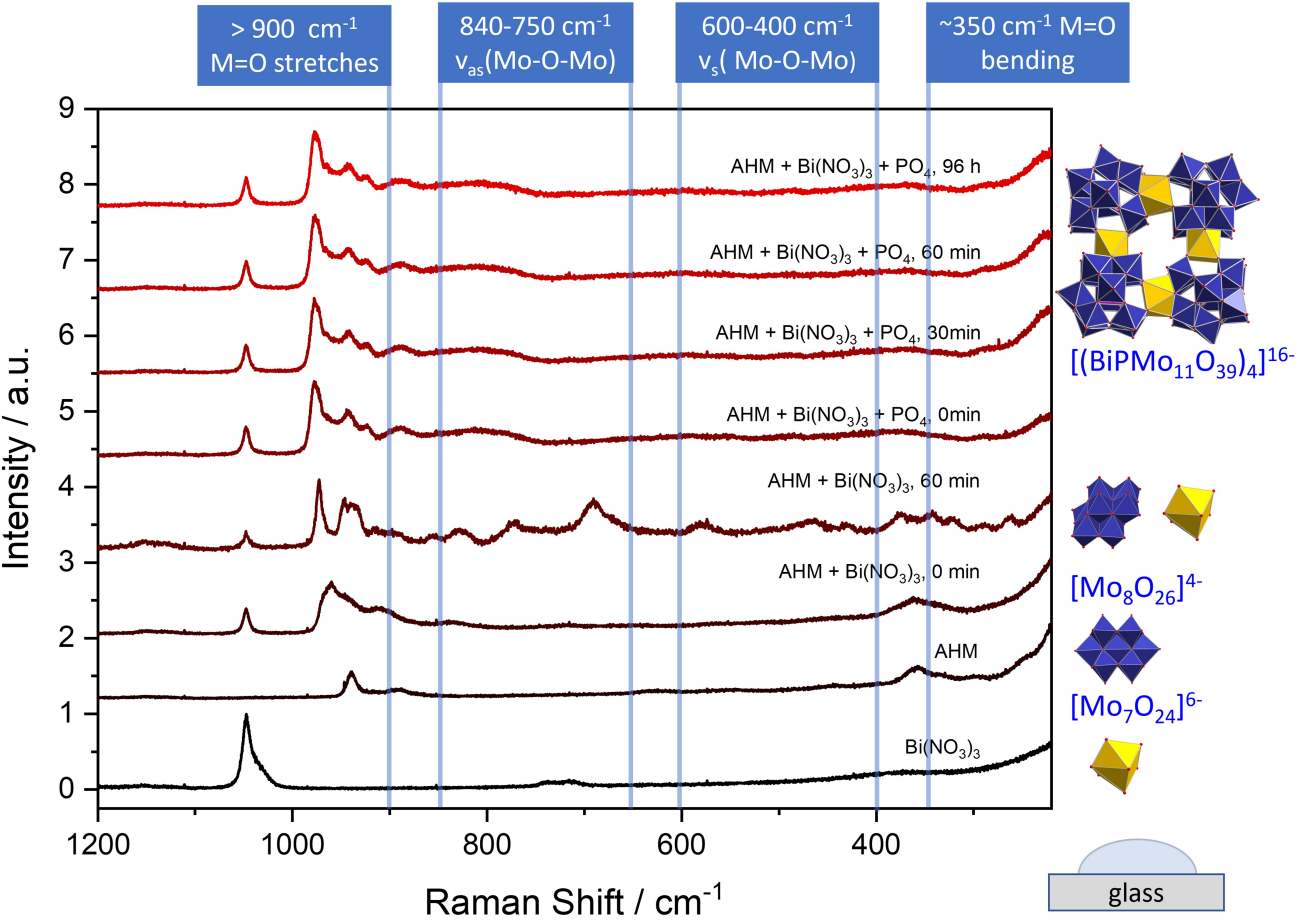
Micro‐Raman spectra obtained during different steps in the synthesis of (P_4_Bi_4_Mo_44_). On the right side different polyoxometalates identified are shown. The spectra were obtained from solutions deposited on a glass slide. A detailed assignment of the bands can be found in the Supporting Information Figures S4 and S5.

The Raman spectrum of an aqueous bismuth nitrate solution shows a characteristic band at 1048 cm^−1^ which can be assigned to NO_3_^−^ anions^[16]^ For the aqueous ammonium heptamolybdate solution typical bands at 938, 892, 630, 554, and 359 cm^−1^ can be assigned to the heptamolybdate anion [Mo_7_O_24_]^6−^ in good agreement with the values in literature.^[17]^ After mixing the starting solutions a decrease of the pH of the solution from pH=5.3 to pH=2.3 is observed. Under these conditions molybdate solutions tend to form molybdate anions with higher condensation degree. Consequently, a new band position of n_s_(Mo‐O_t_) appears at 963 cm^−1^ indicating the increased condensation degree of the molybdate ions. Additionally, the following bands are observed: 946, 907, 836, and 360 cm^−1^ which allow an assignment to [Mo_8_O_26_]^4−^ anions (wavenumbers of overlapping bands were determined by deconvolution into Lorentz peaks,^[18]^ see Figure S5, Supporting Information).^[17a]^

While the most prominent band at 963 cm^−1^ perfectly matches the according wavenumber of γ‐[Mo_8_O_26_]^4−^ described in the literature, the assignment of the observed spectrum to α‐[Mo_8_O_26_]^4−^ (959 cm^−1^) cannot be completely excluded.^[17b]^ After 60 min this Raman band appears shifted to 972 cm^−1^, which is in agreement with the formation of β‐[Mo_8_O_26_]^4−^.^[17b]^ After addition of phosphoric acid the characteristic bands of the final compound are observed: 976, 942, 923, and 890 cm^−1^.^[19]^

The formation mechanism of the Keggin ring in solution was followed using a TRIS approach which comprises EXAFS and SAXS/WAXS experiments.

EXAFS measurements at the Mo−K edge (20 000 eV) and the Bi−L_3_ edge (13 419 eV) were performed in a custom‐made in situ cell allowing monitoring of typical beaker chemistry synthesis under heating with continuous stirring (see Figure 3a). The reactions were repeated under the same conditions at the Mo−K (Figure 3b) and Bi−L_3_ (Figure 3c) edges. In all Mo−K edge XANES region, spectra show clear pre‐edge features characteristic for Mo(VI) which are not observed for lower oxidation states.^[20]^ The edge positions do not shift during the reaction. All Bi L_3_‐edge spectra correspond to Bi(III) oxidation state.^[21]^ The starting solutions differ for the Mo−K edge measurements (AHM solution) and Bi−L_3_‐edge measurements (bismuth nitrate solution). The EXAFS curves of the initial Mo‐containing solution can be fitted with the structure of ammonium heptamolybdate (NH_4_)_6_(Mo_7_O_24_ ⋅ 4H_2_O.^[22]^ This is in accordance with previous studies showing that at pH value below 7 (here pH=5.3), the [Mo_7_O_24_]^6−^ anion is the predominant species in aqueous solution.^[17a]^ All spectra are in agreement with Mo coordinated by six oxygen atoms in its first coordination sphere (Figure 3b). These initial EXAFS curves have characteristic maxima, which correspond to the second coordination sphere between 2.5 and 3.2 Å. The first Bi−L_3_ edge EXAFS curves (see Figure 3c) are characteristic for an acidic bismuth nitrate solution and could be fitted with a structure of tetraoxotetrahydroxobismuth(III) nitrate monohydrate, Bi_6_O_4_(OH)_4_(NO_3_) ⋅ 6 H_2_O.^[23]^ The Bi^3+^ ions are only coordinated by oxygen in a (6+1) coordination sphere.

**Figure 3:**
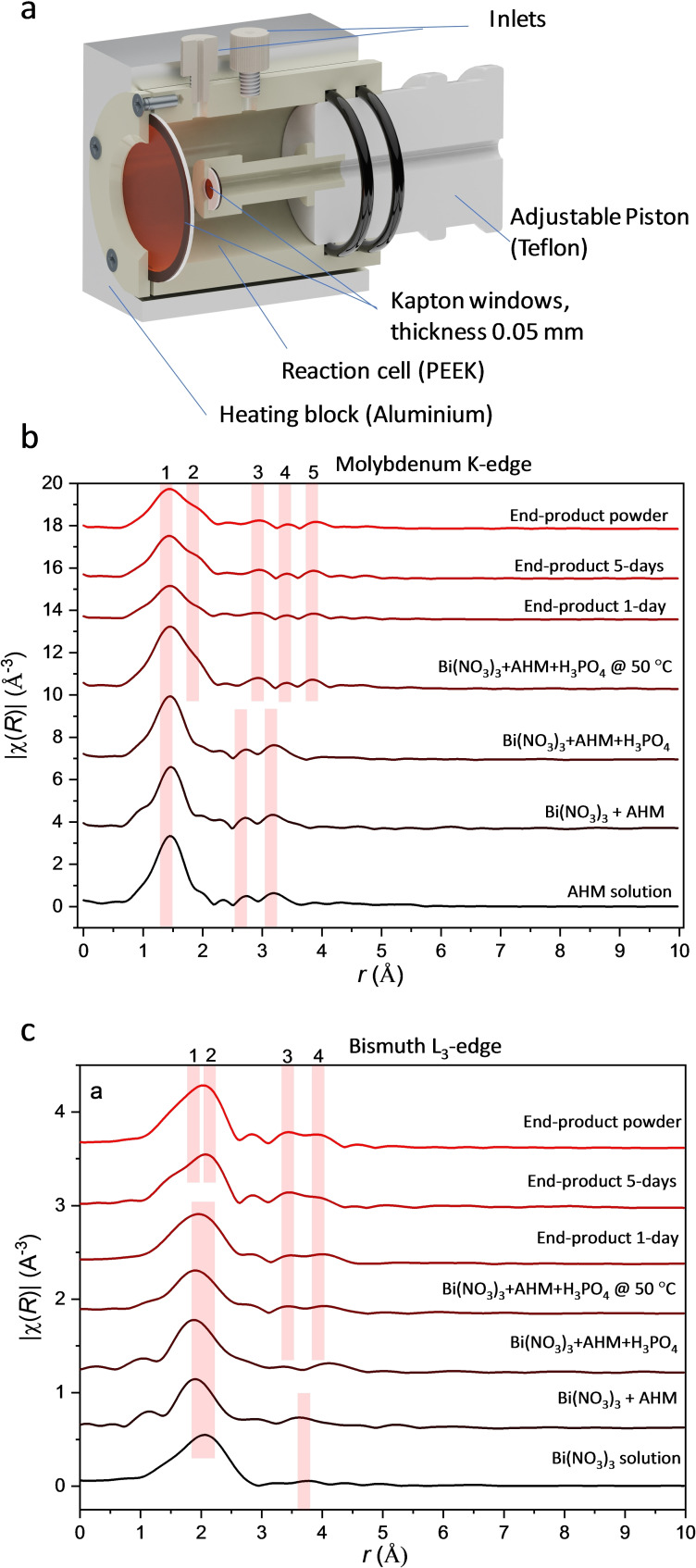
a) Side view of the in situ cell used for the EXAFS experiments. Experimental EXAFS data (from dark red to red) at the Mo−K edge (b) and Bi−L_3_‐edge (c).

After addition of the Bi‐solution a white, cloudy precipitate consisting of large inhomogeneous flakes is obtained and a change in the Mo‐spectrum is observed. The decrease in the pH value (pH=2.3) leads to a condensation of the molybdate anions to [Mo_8_O_26_]^4−^ polyoxomolybdate anions, whereby each [Mo_8_O_26_]^4−^ anion coordinates to Bi^3^ after adding the H_3_PO_4_ solution and upon heating (to 50 °C). the precipitate becomes more homogeneous. In this period a significant change in the Mo coordination is observed. The EXAFS spectra change to the characteristic spectrum of the final ring structure. EXAFS data collected from the final product in solution and in solid‐state can be both fitted with the measured single‐crystal structure.

The corresponding Bi−L_3_ EXAFS data shows significant changes in the Bi coordination. The Bi−O coordination increases from 6 to 8 after heating. The second coordination sphere contains four Mo atoms before heating and 8 Mo neighbours after heating. The EXAFS spectrum of a five‐day old solution could here also be fitted with the structure of the final product. The results of the fitted EXAFS data are summarized in Tables S1 and S2 for Mo−K and Bi−L_3_ edges, respectively (see Supporting Information).

The SAXS/WAXS in situ measurements were performed using a custom‐made flow cell with a 1.5 mm glass capillary in the X‐ray beam (see Figure 4a). Based on the data, the reaction can be divided into four stages according to the presented temperature profile (Figure 4b) and assigned to different crystalline phases (Figure 4c, and Figure S6). The pure AHM solution has a distinct form factor (R_g_∼1 nm) consistent with [Mo_7_O_24_]^6−^ present in the solution. After injection of the bismuth nitrate solution the SAXS signal changes within a single data frame of 10 s. The form factor changes and in addition a 100‐fold increase in intensity at low‐q is observed (see Figure 4d). This indicates aggregation of smaller nanoparticles. This aggregation progresses, but within the first 10 data frames a constant scattering pattern is observed.

**Figure 4:**
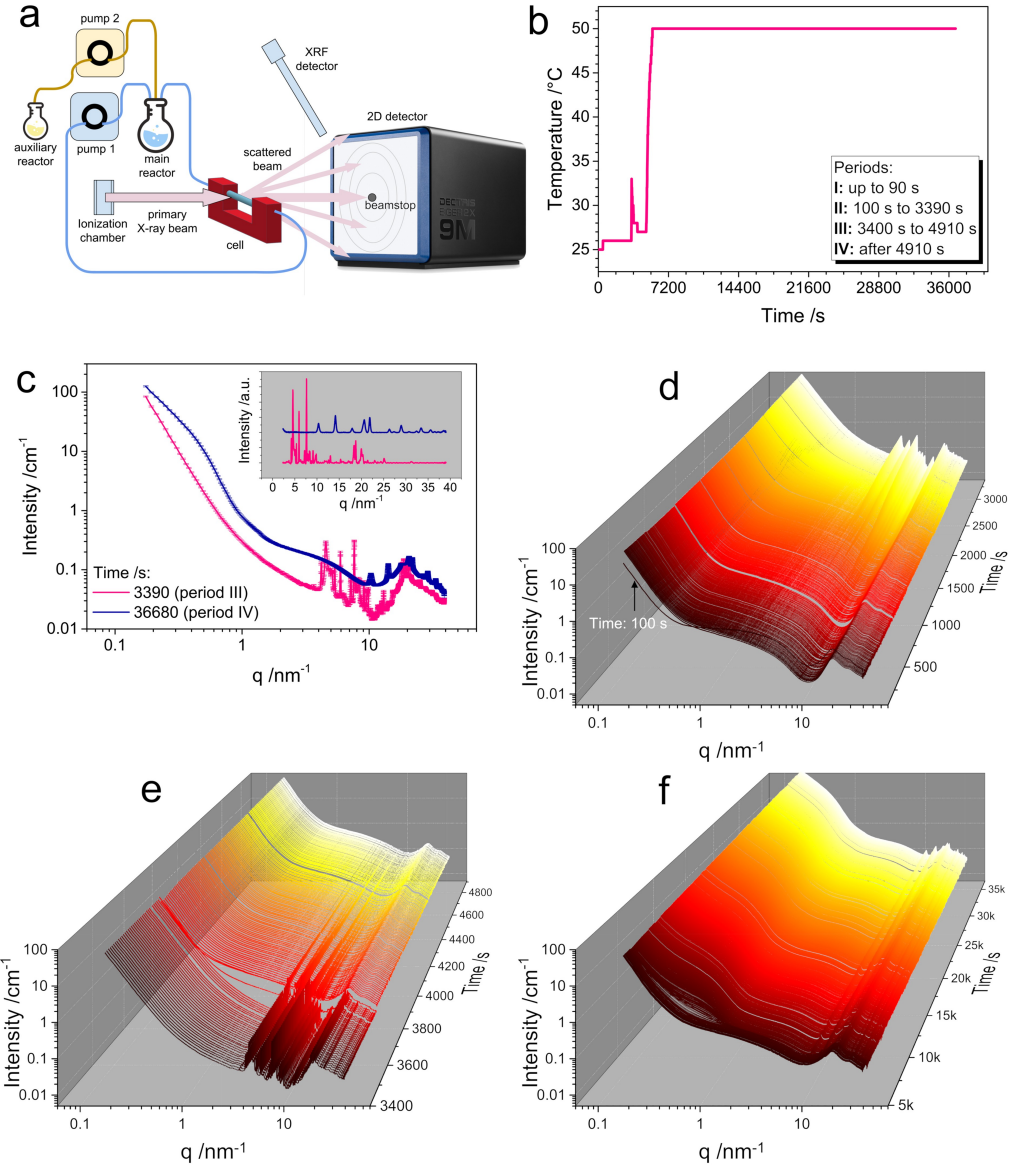
Time‐resolved in situ SAXS/WAXS data for the formation of **(P_4_Bi_4_Mo_44_)** consisting of four stages, a) general scheme of the flow‐setup; b) temperature profile for the synthesis of **(P_4_Bi_4_Mo_44_)**; c) comparison of diffraction patterns obtain at stages III and IV during the formation of **(P_4_Bi_4_Mo_44_)**; d) stage II: time‐dependent scattering curves showing the crystallization of the aggregated particles, forming(NH_4_)_8_(Mo_36_O_112_(H_2_O)_16_)(H_2_O)_37_; e) stage III: after injection of H_3_PO_4_, the crystalline phases dissolve gradually; f) stage IV: crystallization of BiPO_4_ upon heating.as negative control).

This suggests that the evolution at the particulate level stops or slows down significantly. After approx. 1500 s a highly crystalline phase starts to develop. This WAXS pattern could be assigned to a [Mo_36_O_112_]^6−^ unit containing structure (K_8_(Mo_36_O_112_(H_2_O)_16_)(H_2_O)_37_, PDF 73‐2295, in our case NH_4_^+^ is the counter ion) and Bi_6_O_5_(OH)_3_(NO_3_)_5_(H_2_O)_3_ (PDF 70‐1226). After the injection of H_3_PO_4_, the process is highly exothermic according to the T‐profile. During the reaction of the H_3_PO_4_, the crystalline phase gradually dissolves up to approx. 4500 s (see Figure 4e). The SAXS signal decreases, which may suggest deaggregation of particles. During the tempering step the crystallization of BiPO_4_ was observed (Figure 4f).

Evaluating the results of the different analytical methods, one has to bear in mind that different areas of the solution are examined. Raman microscopy for example, probes the solution part whereas the in situ setups for EXAFS and SAXS/WAXS probe both solution and precipitates (Figure 3, 4).

In agreement with previous studies, we could confirm that different isopolyoxomolybdate species (co)exist in equilibrium in solution depending on the pH value (see Figure 5). The formation of the lacunary Keggin ions is triggered by the addition of phosphoric acid.

**Figure 5:**
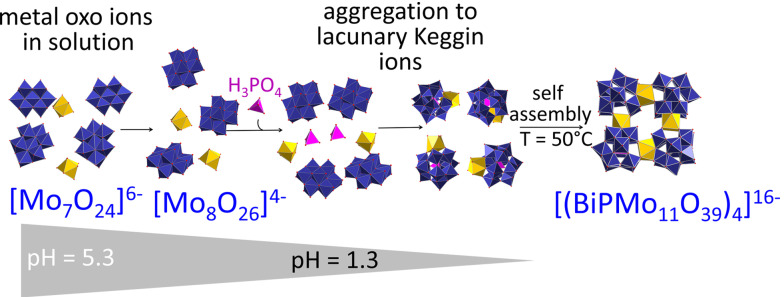
Proposed mechanism for the formation of **(P_4_Bi_4_Mo_44_)**, showing the coordination polymers in solution and the self‐assembly to the final structure at elevated temperature.

The following mechanism of formation of **(P_4_Bi_4_Mo_44_)** is in accordance with the ex situ and in situ data: i) Formation of the lacunary Keggin ion via bismuth insertion and ii) ring closure of the tetrameric ring linking four [α‐PBiMo_11_O_39_]^4−^ together in a final self‐assembly step. Most probably, the substitution of a Mo^6+^ cation by the lower valent Bi^3+^ cation leads to an increase in the basicity of the lacunary Keggin ions and thus a tendency to oligomerize through intermolecular Bi−O…O−Mo contacts). MALDI‐TOF data (Figure S6, Supporting Information) indicate that dimeric structures are formed in between.^[24]^

Alternatively a dimer of dimers mechanism could also lead to a tetrameric structure. However, the final step is difficult to access as can be assumed from both Raman and EXAFS data indicating that the annealed samples already greatly resemble the final structure. Similar conclusions have been drawn by Kortz et al. for tetrameric ring structure [{β‐Ti_2_SiW_10_O_39_}_4_]^24−^.^[25]^ The thermal decomposition of **(P_4_Bi_4_Mo_44_)** was studied by TG‐DSC‐MS (Figure S7) analysis and temperature‐resolved XRD (Figure S8). The lacunary anion complex is destroyed above 250 °C forming a crystalline cubic phase containing intact Keggin ions ((NH_4_)_3_PO_4_(MoO_3_)_12_ ⋅ 4 H_2_O). This phase decomposes above 450 °C under formation of BiPO_4_ and MoO_3_.

In summary, we report on the synthesis, structural characterization, and formation mechanism of a new polyoxometalate ring structure composed of lacunary Keggin ions connected via Bi cations. The structure represents the first example of a tetrameric bismuth‐molybdate nano‐ring. We investigated the formation process using time‐resolved in situ EXAFS and in situ SAXS/WAXS complemented by Raman spectroscopy and MALDI‐TOF spectroscopy. The formation of the tetrameric anion under mild conditions indicates that Bi^3+^‐substituted polyoxomolybdates are prone to form larger cyclic entities. More broadly, our study shows that the incorporation of Bi can offer access to larger ring structures.

## Experimental Section

See Supporting Information for full details of synthesis, characterisation methods and physical measurements.

Deposition Number(s) 2124803 (for **P**_**4**_ **Bi**_**4**_ **Mo**_**44**_) contain(s) the supplementary crystallographic data for this paper. These data are provided free of charge by the joint Cambridge Crystallographic Data Centre and Fachinformationszentrum Karlsruhe Access Structures service www.ccdc.cam.ac.uk/structures.

## Conflict of interest

The authors declare no conflict of interest.

## Supporting information

As a service to our authors and readers, this journal provides supporting information supplied by the authors. Such materials are peer reviewed and may be re‐organized for online delivery, but are not copy‐edited or typeset. Technical support issues arising from supporting information (other than missing files) should be addressed to the authors.

Supporting Information

## Data Availability

The data that support the findings of this study are available from the corresponding author upon reasonable request.
