# Supplementary material for: Formation Mechanism of a Nano‐Ring of Bismuth Cations and Mono‐Lacunary Keggin‐Type Phosphomolybdate
Source: Chemistry. 2022 Apr 1;28(27):e202200079. doi: 10.1002/chem.202200079 (PMC9322599; doi:10.1002/chem.202200079)
Supplement: Supplementary file 1 — Supporting Information [file CHEM-28-0-s001.pdf]

Chemistry–A European Journal

Supporting Information

Formation Mechanism of a Nano-Ring of Bismuth Cations and Mono-Lacunary Keggin-Type Phosphomolybdate

Inês C. B. Martins, Dominik Al-Sabbagh, Ursula Bentrup, Julien Marquardt, Thomas Schmid, Ernesto Scoppola, Werner Kraus, Tomasz M. Stawski, Ana Guilherme Buzanich, Kirill V. Yusenko, Steffen Weidner, and Franziska Emmerling*

Abstract: A new hetero-bimetallic polyoxometalate (POM) nano ring was synthesized in a one-pot procedure. The structure consists of tetrameric units containing four bismuth-substituted monolacunary Keggin anions including distorted [BiO8] cubes. The nano ring is formed via self-assembly from metal precursors in aqueous acidic medium. The compound $(NH_4)_{16}[(BiPMo_{11}O_{39})_4]\cdot 22H_2O$; **($P_4Bi_4Mo_{44}$)** was characterized by single-crystal X-ray diffraction, extended X-ray absorption fine structure spectroscopy (EXAFS), Raman spectroscopy, matrix-assisted laser desorption/ionisation-time of flight mass spectrom-etry (MALDI-TOF), and thermogravimetry/differential scanning calorimetry (TG-DSC-MS). The formation of the nano ring in solution was studied by time-resolved *in situ* small- and wide-angle X-ray scattering (SAXS/WAXS) and *in situ* EXAFS measurements at the Mo-K and the Bi-L_3 edge indicating a two-step process consisting of condensation of Mo-anions and formation of Bi-Mo-units followed by a rapid self-assembly to yield the final tetrameric ring structure

1. Materials and Methods 3
Synthesis 3
Raman spectroscopy 3
MALDI-TOF3
TG-DSC-MS 4
Powder X-ray diffraction 4
Single crystal X-ray diffraction 4
In situ SAXS/WAXS 5
In situ EXAFS 6
2. Crystal structure 8
3. Physical measurements 11
Raman data evaluation 11
EXAFS data evaluation 14
SAXS/WAXS curves different periods 19
MALDI TOF data 20
TG-DSC-MS, ATR-IR analysis, and temperature-depended XRD 21
References 23

1 1. Materials and Methods

1.1 Synthesis

The bismuth containing aqueous slurry was prepared by subsequent adding of bismuth(III) nitrate pentahydrate/HNO_3 solution and phosphoric acid (H_3PO_4) to an ammonium heptamolybdate tetrahydrate ($(NH_4)_6Mo_7O_{24}\cdot 4H_2O$, AHM) solution. In the first step the bismuth(III) nitrate pentahydrate (1.944 g, 4 mmol; 98 %, Alfa Aesar) was poured into a HNO_3 solution (0.5mL 65% HNO_3 in 5mL H_2O) and then dropwise added to the AHM solution (8.472 g, 6.86 mmol in 30 mL H_2O; 99%, Chemsolute) at room temperature under continuous stirring. Afterwards, this mixture was stirred for 1 h at 25 °C. In the second step 0.5 mL of H_3PO_4 (85%, Acros Organics) were added. The reaction mixture was stirred for 30 min at 25 °C, 24 h at 50°C and subsequently 5 days at 25 °C. The orange slurry was filtered and washed 3 times with 20 mL deionized water, followed by air drying. Suitable crystals for single crystal X-ray analysis were obtained during the drying step.

1.2 Raman spectroscopy

The Raman spectra were acquired by using a LabRam HR 800 instrument (Horiba Jobin Yvon) coupled to a BX41 microscope (Olympus). For both excitation and collection of the scattered light, a 50x/NA = 0.55 objective lens was employed. The system is equipped with a diode laser that has a wavelength of 785 nm and 1800 mm^{-1} grating. The spectrometer entrance slit was 100 µm wide and the confocal pinhole was in the fully open position (1000 µm). In this configuration the instrument provides a spatial resolution of approx. 870 nm laterally and approx. 75 µm along the optical axis, while the spectral resolution within the observed spectral range amounts to approx. 0.15 cm^{-1}.[1] The spectra were acquired with a charge-coupled device camera (Syncerity CCD, Horiba Jobin Yvon) operating at -60 °C (Deep Thermoelectric Cooling). Each spectrum was accumulated over two individual measurements, each with an acquisition time of 30 seconds and a laser power of approx. 40 mW at the sample surface (100% of the available laser power). Prior to the Raman experiment, the laser was allowed to stabilize for at least 2 h and the spectrometer was recalibrated against the most prominent mode of silicon at 520.7 cm^{-1}. The spectra were smoothed by applying a moving average over three measurement points and were scaled and stacked by adding offset values for better comparability. For determining exact band positions, groups of overlapping bands were deconvoluted by fitting with linear combinations of Lorentz functions, as previously described in Ref.[2]

1.3 **MALDI-TOF**

The MALDI-TOF mass spectra were obtained on a Bruker Daltonics MALDI AutoflexMax spectrometer in positive linear mode with delayed extraction (150 ns). The instrument is equipped with a Nd-YAG laser (355 nm) with a frequency of 2000 Hz. The mass spectra were recorded by accumulation of 8000 laser shots recorded at four different

spot regions. The sample was prepared by applying the so-called solventless sample preparation procedure. For this, 10 mg of sample were mixed with 50 mg of matrix ([3-(4-tert-Butylphenyl)-2-methyl-2-propenylidene]malononitrile – DCTB) in a 0.5 mL polypropylene vial containing five stainless steel balls of 1 mm were added. The vial was vortexed for 1 min using a Vortex Genie-2 (Scientific Instruments, USA). The resulting powder mixture was deposited on the MALDI target holder using a spatula.

Calibration was performed externally using the Bruker Daltonics peptide calibration standard in a mass range between *m/z* ~1000 and 10000.

1.4 **TG-DSC-MS**

The measurements were performed on a Sensys TG-DSC apparatus (Setaram) coupled with a OmniStar quadrupole mass spectrometer (Pfeiffer Vacuum). The sample (44.47 mg, Al_2O_3 crucible) was heated in synthetic air (20 ml/min) with 5 K/min.

ATR-IR

ATR-IR spectra of the solid samples were measured on an ALPHA FTIR spectrometer (Bruker).

1.5 **Powder X-ray diffraction**

Powder X-ray diffraction (PXRD) measurements were performed in Bragg-Brentano geometry in a 2θ range from 4° to 80°, with a step size of 0.005° (D8 Advance, Bruker AXS, Germany). The diffraction patterns were collected using Cu Kα (λ_1 = 1.54056 Å, λ_2 = 1.54443 Å) radiation. The diffraction patterns were recorded with a Lynxeye-XET position sensitive detector at room temperature. Si powder (NIST SRM 640, 5.431179(8) Å) was used as external standard.

Figure S2 shows a comparison of the measured and calculated PXRD data of **($P_4Bi_4Mo_{44}$)**.

Temperature dependent X-ray diffraction measurements were conducted using the modular temperature chamber for D8 Advance within a temperature range from 25 to 545°C in 10K steps. To guaranty temperature stability, the temperature was kept for 10min prior to each measurement. The sample displacement was minimized by height adjustment using corundum powder. Furthermore, the corundum powder was measured for the same parameters and the captured diffraction pattern was used for the background correction of all recorded diffraction data.

1.6 Single crystal X-ray diffraction

The diffraction data were collected on a Bruker APEX-II CCD diffractometer using Mo Kα radiation. Data collection and data reduction were performed using the APEX2 and

SAINT software.[3] The correction for polarization, Lorenz and absorption was performed using the SADABS program. The structure was solved by direct methods and afterwards refined by full-matrix least squares on F^2 using the SHELXTL software.[4] All non-hydrogen atoms were refined anisotropically. The determination of the exact position of the hydrogen atoms from water and ammonium ions was not possible. Therefore, the most probable positions for the hydrogen atoms were calculated by fast DFT in Avogadro program and refined using the SHELXTL software.[4] However, short hydrogen-hydrogen interactions were detected and therefore the hydrogen atoms were not included in the final structure. Selected bond lengths and angles are listed in Tables S1 and S2. CCDC-2124803 contains the supplementary crystallographic data for **($P_4Bi_4Mo_{44}$)**.

1.7 In situ SAXS/WAXS

Combined small- and wide-angle X-ray scattering (SAXS/WAXS) measurements were performed on a µSpot[5] beamline of BESSY-II (Helmholtz-Zentrum Berlin, HZB, Germany). Experiments were performed using a monochromatic X-ray beam at 15.0 keV aligned with a capillary of the flow-through setup and scattered intensities were collected at small- and wide-angles with a Dectris Eiger 9M detector. Transmission through the sample was calculated from an X-ray fluorescence signal collected from a lead beamstop using RAYSPEC Sirius SD-E65133-BE-INC detector equipped with an 8µm beryllium window, where the primary beam intensity (I0) was monitored and normalised using an ion chamber. The sample-to-detector distance of ~330 mm allowed for a usable q-range of $\sim 0.1 < q < 35$ nm^{-1}. The scattering q-range was calibrated against silicon powder (NIST SRM640) and the corresponding measured intensity was calibrated to absolute units against water (at 25°C) and glassy carbon (NIST SRM3600). The resulting data were pre-processed/previewed using the DPDAK software package[6], where the actual processing was performed in custom Python scripts utilizing a pyfai library.[7] The pre-processing steps involved integration to 1D scattering curves and subtraction of an instrumental background (*i.e.* an “empty beamline” or “air” background). The scattering data were corrected for transmission and primary beam intensity and scaled to absolute intensity units. The as-obtained raw scattering curves were further corrected for a “container background” (see below).

The scattering data from the formation of phosphomolybdate Keggin clusters were measured in multiple cycles, where each cycle included an “empty beamline” measurement for 10 s followed by 40 frames of 10 s each from the sample. In the course of the experiment, this allowed us to collect a large number of up to date “beamline” backgrounds, which were used to correct the time-resolved data for air scattering, and to monitor any potential variations in beamline settings. An empty cell scattering was used as a “container background”. The diffraction peaks were extracted from the scattering data by applying an Asymmetric Least Square Smoothing baseline correction method.[8]

Phosphomolybdate precipitation was performed in a 200 mL temperature-stabilized glass reactor equipped with a passively-cooled reflux condenser to prevent any evaporation of solvents (primary reactor in Fig. 4). The reaction temperature in the vessel was maintained by a computer-controlled hot plate with a thermocouple placed directly in the solution. The reacting solutions were continuously stirred at 1000 rpm and circulated through a custom-built PEEK flow-through cell with an embedded borosilicate glass capillary (ID 1.5 mm) using a peristaltic pump (flow 3 mL/second). The synthesis followed exactly the same stages as those described above and was started by circulating the AHM solution through the capillary cell. Injection and mixing of the reactants was performed by means of a secondary peristaltic pump (flow 3 mL/second) as a remote injection system, which was connected to an auxiliary reactor.

1.8 In situ EXAFS

The experiments were performed in a custom-made *in situ* cell allowing to monitor typical beaker chemistry synthesis under continuous stirring using X-ray beam in transmission or in reflection (Figure S1). Our *in situ* cell with a reaction volume up to 15 mL (min volume is about 10 mL) has two Kapton windows for primary and secondary beams and possibility to stir and heat reaction volume. Special holes on the top of the cell can be used for temperature control as well as for adding reactants using automatic syringe pump or manually. An Aluminum block is used for better heat exchange between stirring hot plate and reaction cell. Reaction cell is sealed and can be used up to 170-180 °C with inert and reactive solutions. Heating and stirring is also controlled remotely. To adopt beam path for EXAFS measurements movable piston as the first window is used. A movable piston allows to change beam path between 1 mm up to 20-40 mm. All parts are easy to exchange in case of their damage. Especial attention should be paid to X-ray flux damage of Kapton windows.

EXAFS measurements were performed in transmission at the BAMline located at the BESSY II facility in Berlin.[9] Energy was scanned by a Si(111) double crystal monochromator in steps of 10 eV before the edge, 1 eV until 200 eV above the edge and in 0.04 Å equidistant k-steps in the extended region for EXAFS. A measurement time of 2 seconds per point was applied, allowing a good S/N ratio. The data was normalized using ATHENA and EXAFS fits using model structures by FEFF in ARTEMIS, all from IFEFFIT package software.[10]

Interatomic distances fitted based on EXAFS for Mo-K-edge and Bi-L_3-edge data are presented in Tables S3 and S4.

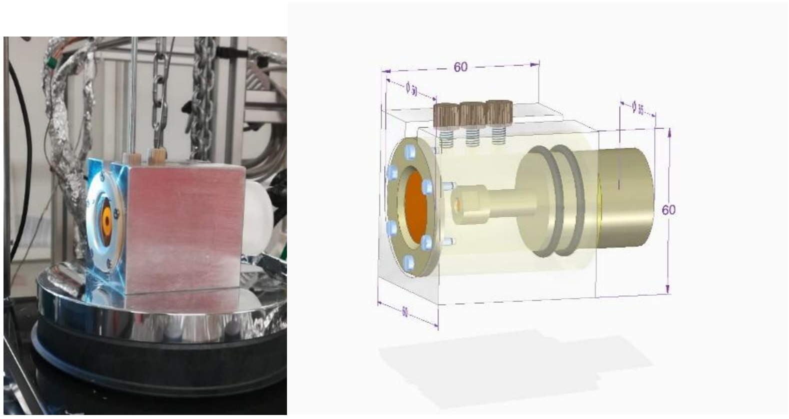

Figure S1 Synchrotron setup of the reaction cell on the stirring hot plate (left). Technical drawing of in situ reaction cell for transmission and reflection X-ray spectroscopy and diffraction studies (right).

2. Crystal structure

The crystal structure of **($P_4Bi_4Mo_{44}$)** consists of an aggregate anion $[Bi_4P_4Mo_{44}O_{156}]_2^{16-}$ constructed from four identical monolacunary Keggin-type fragments ($[\alpha\text{-}PMo_{11}O_{39}Bi]^{4-}$). The resulting ring structure consists of a ring with a size of 2.3 x 2.3 x 1.4 nm^3. This ring structure is surrounded by twelve water and sixteen ammonium molecules.

Bi-O bond distances range from 2.3221(44) to 2.4949(72) Å and a comparable to known heteropolytungstates exhibiting an 8-fold square - antiprismatic coordination geometry.[11] [12] The Mo-O bond distances range from 1.6831(77) to 2.499 (6) Å. Along the *a*-axis in the unit cell the **($P_4Bi_4Mo_{44}$)** rings are stacked parallel with an inclined angle of 47° of the ring plane with respect to the *b*-axis (see Figure S3). The ring planes of next layer of **($P_4Bi_4Mo_{44}$)** rings in *b* direction are tilted by nearly 90° (84.29°) and stacked in a herringbone pattern.

Deposition Number CSD-2124803 contain(s) the supplementary crystallographic data for this paper. These data are provided free of charge by the joint Cambridge Crystallographic Data Centre and Fachinformationszentrum Karlsruhe (http://www.ccdc.cam.ac.uk/structures) Access Structures service.

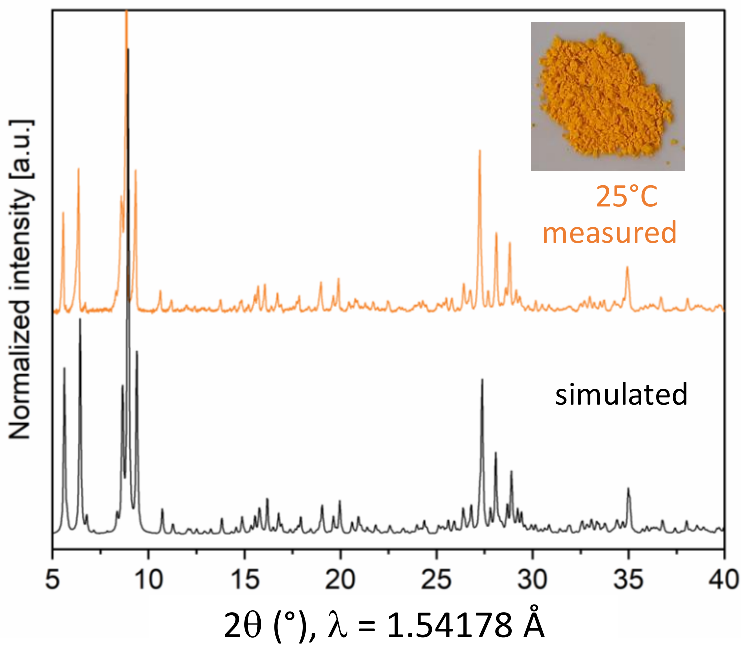

Figure S2. Experimental X-ray diffraction powder pattern of **($P_4Bi_4Mo_{44}$)** (orange) compared to the theoretical pattern calculated from the crystal structure (black).

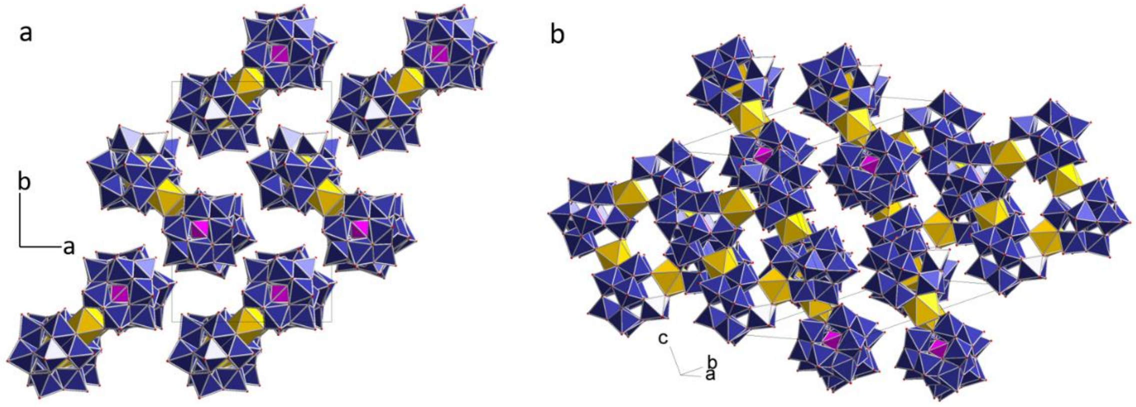

Figure S3. Crystal structure of **($P_4Bi_4Mo_{44}$)** viewed along the c axis showing the perpendicular arrangement of the nano rings. The polyanions are represented polyhedrally; Bi (yellow), Mo (blue), P (pink). The ammonium ions and solvent water molecules are omitted for clarity.

Table 1. Crystal data and structure refinement for **($P_4Bi_4Mo_{44}$)**.

Empirical formula	$(NH_4)_{16}[(BiPMo_{11}O_{39})_4]\cdot 22H_2O$
Formula weight	8253.32
Temperature	293(2) K
Wavelength	0.71073 Å
Crystal system	monoclinic
Space group	P 2_1/n
Unit cell dimensions	a = 17.3264(9) Å
	b = 24.7046(12) Å β= 107.849(2)°.
	c = 21.4084(12) Å
Volume	8722.6(8) Å^3
Z	2
Density (calculated)	3.142 Mg/m^3
Absorption coefficient	7.221 mm^{-1}
F(000)	7552
Crystal size	0.37 x 0.22 x 0.20 mm3
Theta range for data collection	2.060 to 28.738°.
Reflections collected	156546
Independent reflections	22581 [R(int) = 0.1368]
Completeness to theta = 25.242°	99.9 %
Absorption correction	Semi-empirical from equivalents

Max. and min. transmission	0.4275 and 0.2839
Refinement method	Full-matrix least-squares on F2
Data / restraints / parameters	22581 / 0 / 1177
Goodness-of-fit on F^2	1.232
Final R indices [I>2sigma(I)]	R_1 = 0.0479, wR_2 = 0.1184
R indices (all data)	R_1 = 0.0550, wR_2 = 0.1242
Largest diff. peak and hole	2.644 and -2.046 e.Å^{-3}

2 3. Physical measurements

2.1 Raman data evaluation

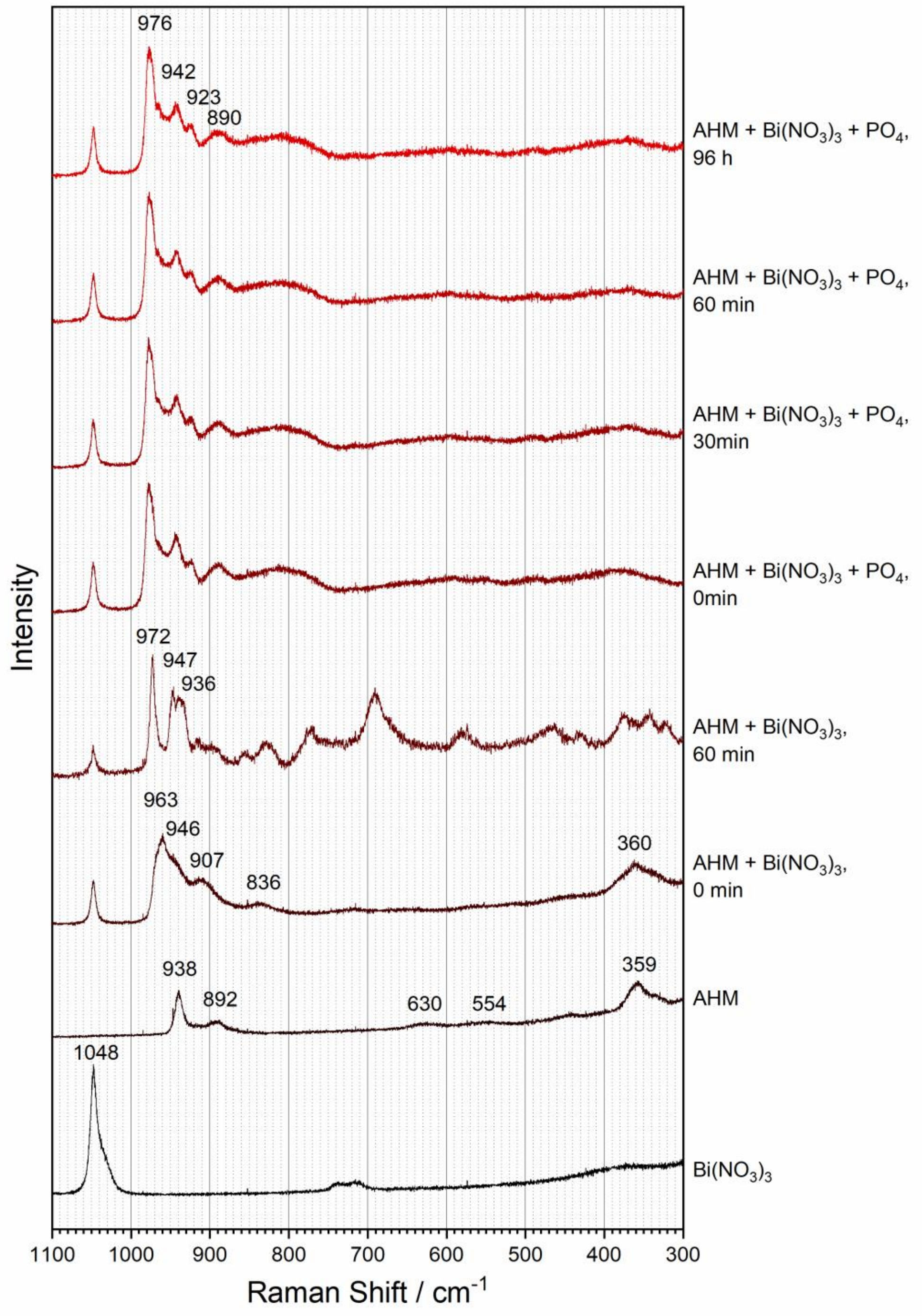

Figure S4. Enlarged view of the Raman spectra shown in Figure 3 in the main text. The bands mentioned in the text are labelled,

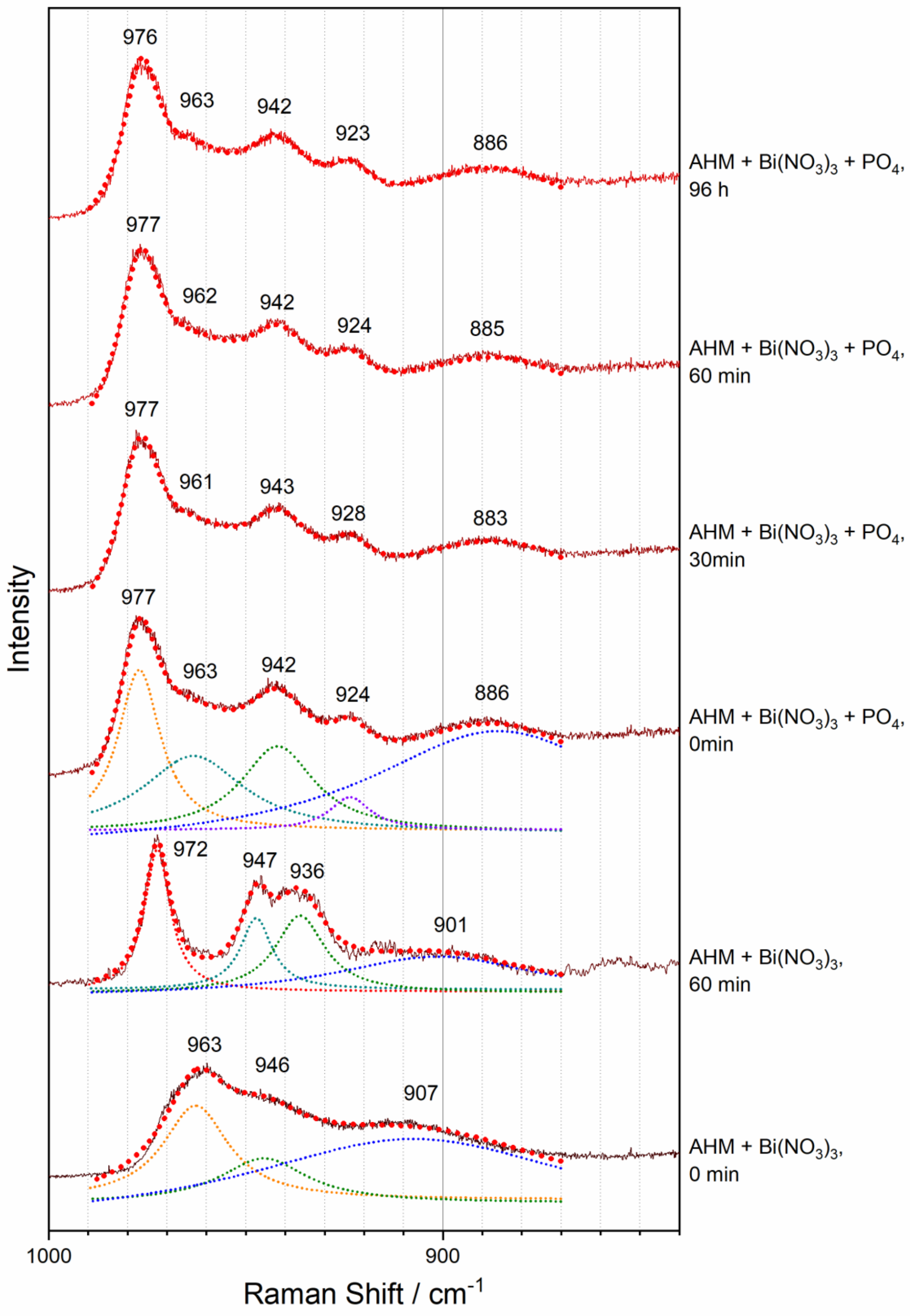

Figure S5. Deconvolutions of Raman spectra in the range of 870 cm^{-1} to 990 cm^{-1} into individual Lorentz peaks.

2.2 EXAFS data evaluation

Table S3. Interatomic distances fitted based on EXAFS Mo-K-edge spectra.

Sample	ed. Chi^2	R-factor	Model: Path	N	R_{eff} (model)	R_{fit} (calculated)	DR	RMSE
starting water solution	44	0.0097	Mo_7O_{24}: Mo-O(3.1)		1.71590	1.66912	-0.04678	0.00618
			Mo_7O_{24}: Mo-O(7.1)		1.76950	1.72272	-0.04678	0.00618
			Mo_7O_{24}: Mo-O(15.1)		1.90810	1.86132	-0.04678	0.00618
			Mo_7O_{24}: Mo-O(20.1)		1.94100	1.89422	-0.04678	0.00618
			Mo_7O_{24}: Mo-O(23.1)		2.15610	2.05825	-0.09785	0.02404
			Mo_7O_{24}: Mo-O(22.1)		2.20080	2.10295	-0.09785	0.02704
			Mo_7O_{24}: Mo…Mo(5.1)	.5	3.17890	3.18587	0.00697	0.01095
			Mo_7O_{24}: Mo…Mo(4.1)		3.24180	3.24877	0.00697	0.01095
			Mo_7O_{24}: Mo…Mo(7.1)		3.36380	3.37077	0.00697	0.01095
$(NH_4)_6Mo_7O_{24}\cdot4H_2O$ + $Bi(NO_3)_3$ solution	59	0.02						
			Mo_8O_{26}: Mo-O(7.1)		1.70450	1.70254	-0.00196	0.00668
			Mo_8O_{26}: Mo-O(8.1)		1.90580	1.90384	-0.00196	0.00668
			Mo_8O_{26}: Mo-O(6.1)		1.99090	1.98894	-0.00196	0.00668
			Mo_8O_{26}: Mo-O(1.1)		2.31330	2.24926	-0.06403	0.01517
			Mo_8O_{26}: Mo-O(6.2)		2.34560	2.28157	-0.06403	0.01517
			Mo_8O_{26}: Mo…Mo(3.1)		3.19580	3.24680	0.05100	0.01354
			Mo_8O_{26}: Mo…Mo(2.1)		3.25810	3.30910	0.05100	0.01354
			Mo_8O_{26}: Mo…Mo(1.1)		3.41320	3.46420	0.05100	0.01354

Sample	ed. Chi^2	R-factor	Model: Path	N	R_{eff} (model)	R_{fit} (calculated)	DR	RMSE
			Mo_8O_{26}: Mo…Mo(3.2)		3.53100	3.58200	0.05100	0.01354
$Bi(NO_3)_3$ + HNO_3 + $(NH_4)_6Mo_7O_{24}$·$4H_2O$ + H_3PO_4 (RT, 1 h)	5	0.0099	$(NH_4)_{16}[Bi_2P_2Mo_{22}O_{39}]_2$ $12H_2O$: Mo-O(26.1)		1.67790	1.65037	-0.02753	0.00526
			Mo_7O_{24}: Mo-O(7.1)		1.76950	1.74197	-0.02753	0.00526
			Mo_7O_{24}: Mo-O(20.1)		1.94100	1.91347	-0.02753	0.00526
			Mo_7O_{24}: Mo-O(23.1)		2.15610	2.18871	0.03261	0.02663
			Mo_7O_{24}: Mo-O(22.1)		2.20080	2.23341	0.03261	0.02663
			Mo_7O_{24}: Mo…Mo(5.1)	.5	3.17890	3.22385	0.04495	0.01157
			Mo_7O_{24}: Mo…Mo(4.1)		3.24180	3.28675	0.04495	0.01157
			Mo_7O_{24}: Mo…Mo(7.1)		3.36380	3.40875	0.04495	0.01157
			Mo_7O_{24}: Mo…O(17.1)		3.76700	3.81195	0.04495	0.01157
$Bi(NO_3)_3$ + HNO_3 + $(NH_4)_6Mo_7O_{24}$·$4H_2O$ + H_3PO_4 (50°C, 1 h)	87	0.014	$(NH_4)_{16}[Bi_2P_2Mo_{22}O_{39}]_2$ $12H_2O$: Mo-O(26.1)	.5	1.67790	1.69054	0.01264	0.01020
			$(NH_4)_{16}[Bi_2P_2Mo_{22}O_{39}]_2$ $12H_2O$: Mo-O(1.1)	.5	1.84610	1.85874	0.01264	0.01020
			$(NH_4)_{16}[Bi_2P_2Mo_{22}O_{39}]_2$ $12H_2O$: Mo-O(4.1)		1.94420	1.95684	0.01264	0.01020
			$(NH_4)_{16}[Bi_2P_2Mo_{22}O_{39}]_2$ $12H_2O$: Mo-O(60.1)		2.26120	2.27384	0.01264	0.01020
			$(NH_4)_{16}[Bi_2P_2Mo_{22}O_{39}]_2$ $12H_2O$: Mo-O1.1…O60.1…O1.1		2.58000	2.38177	-0.19823	0.03537

Sample	ed. Chi^2	R-factor	Model: Path	N	R_{eff} (model)	R_{fit} (calculated)	DR	RMSE
			$(NH_4)_{16}[Bi_2P_2Mo_{22}O_{39}]_2$ $12H_2O$: Mo…Mo(5.1)	.5	3.40450	3.13109	-0.27341	0.01886
			$(NH_4)_{16}[Bi_2P_2Mo_{22}O_{39}]_2$ $12H_2O$: Mo…Bi(1.1)		3.47550	3.20209	-0.27341	0.01886
			$(NH_4)_{16}[Bi_2P_2Mo_{22}O_{39}]_2$ $12H_2O$: Mo…Mo(7.1)		3.72740	3.45399	-0.27341	0.01886
			$(NH_4)_{16}[Bi_2P_2Mo_{22}O_{39}]_2$ $12H_2O$: Mo…Mo7.1…Mo18.1		4.20030	4.32939	0.12909	0.02043
$(NH_4)_{16}[Bi_2P_2Mo_{22}O_{39}]_2$ $12H_2O$ (end product, 1 day at RT)	25	0.015	$(NH_4)_{16}[Bi_2P_2Mo_{22}O_{39}]_2$ $12H_2O$: Mo-O(26.1)		1.67790	1.70373	0.02583	0.01255
			$(NH_4)_{16}[Bi_2P_2Mo_{22}O_{39}]_2$ $12H_2O$: Mo-O(1.1)		1.84610	1.87193	0.02583	0.01255
			$(NH_4)_{16}[Bi_2P_2Mo_{22}O_{39}]_2$ $12H_2O$: Mo-O(4.1)		1.94420	1.97003	0.02583	0.01255
			$(NH_4)_{16}[Bi_2P_2Mo_{22}O_{39}]_2$ $12H_2O$: Mo-O(3.1)		1.99890	2.02473	0.02583	0.01255
			$(NH_4)_{16}[Bi_2P_2Mo_{22}O_{39}]_2$ $12H_2O$: Mo-O(60.1)		2.26120	2.29039	0.02919	0.00288
			$(NH_4)_{16}[Bi_2P_2Mo_{22}O_{39}]_2$ $12H_2O$: Mo…Mo(5.1)		3.40450	3.09632	-0.30818	0.01718
			$(NH_4)_{16}[Bi_2P_2Mo_{22}O_{39}]_2$ $12H_2O$: Mo…Bi1.1		3.47550	3.16731	-0.30818	0.01718

Sam-<br>ple	ed.<br>Chi^2	R<br>-factor	Model:<br>Path	N	R<br>$_{eff}$ (model)	R<br>$_{fit}$ (cal-culated)	D<br>R	R<br>MSE
			$(NH_4)_{16}$ $[Bi_2P_2Mo_{22}O_{39}]_2$ $12H_2O$: Mo…Mo(7.1)		3<br>.7274	3<br>.41922	-<br>0.30818	0<br>.01718
			$(NH_4)_{16}$ $[Bi_2P_2Mo_{22}O_{39}]_2$ $12H_2O$: Mo…Mo7.1…Mo18.1		4<br>.20030	4<br>.31211	0<br>.11181	0<br>.02088

Table S4. Interatomic distances fitted based on EXAFS Bi-L_3-edge spectra.

Sample	R<br>ed. Chi^2	R<br>-factor	Model:<br>Path	N	R<br>$_{eff}$ (model), Å	R<br>$_{fit}$ (calcu-lated), Å	D<br>R, Å	R<br>MSE, Å
$Bi(NO_3)_3$ water solution with HNO_3	1.7	0<br>.01016	$Bi_6O_4(OH)_4(NO_3)$ $6H_2O$: Bi-O(7.1)		2<br>.38400	2<br>.38414	0<br>.00014	0<br>.02014
			$Bi_6O_4(OH)_4(NO_3)$ $6H_2O$: Bi-O(1.1)		2<br>.13620	2<br>.13634	0<br>.00014	0<br>.02014
			$Bi_6O_4(OH)_4(NO_3)$ $6H_2O$: Bi-O(26.1)		3<br>.02780	3<br>.00	-<br>0.02403	0<br>.03970
			$Bi_6O_4(OH)_4(NO_3)$ $6H_2O$: Bi…Bi(6.1)		3<br>.65600	3<br>.63	-<br>0.02403	0<br>.03970
			$Bi_6O_4(OH)_4(NO_3)$ $6H_2O$: Bi…Bi(1.1)		3<br>.69210	3<br>.67	-<br>0.02403	0<br>.03970
$Bi(NO_3)_3$ + HNO_3 + $(NH_4)_6Mo_7O_{24}\cdot 4H_2O$ + H_3PO_4 (RT, 1 h)	48.6	0<br>.016	$Bi_6O_4(OH)_4(NO_3)$ $6H_2O$: Bi-O(1.1)		2<br>.13620	2<br>.29621	0<br>.16001	0<br>.02424
			$(NH_4)_{16}$ $[Bi_2P_2Mo_{22}O_{39}]_2$ $12H_2O$: Bi…Mo(10.1)		3<br>.87740	3<br>.94800	0<br>.07060	0<br>.02017
			$(NH_4)_{16}$ $[Bi_2P_2Mo_{22}O_{39}]_2$ $12H_2O$: Bi…O(28.1)		3<br>.90760	4<br>.06761	0<br>.16001	0<br>.02424
			$(NH_4)_{16}$ $[Bi_2P_2Mo_{22}O_{39}]_2$		4<br>.15560	4<br>.22620	0<br>.16001	0<br>.02017

Sample	ed.<br>Chi^2	R<br>-factor	Model:<br>Path	N	R<br>$_{eff}$ (model), Å	R<br>$_{fit}$ (calcu-lated), Å	D<br>R, Å	R<br>MSE, Å
			$12H_2O$:<br>Bi…O(14.1)					
			$(NH_4)_{16}$<br>$[Bi_2P_2Mo_{22}O_{39}]_2$<br>$12H_2O$:<br>Bi…O(10.1)		2<br>.35840	2<br>.51841	0<br>.0.1600	0<br>.02424
$Bi(NO_3)_3$ + HNO_3 + $(NH_4)_6Mo_7O_{24}$·4 H_2O + H_3PO_4 (50 °C, 18 h)	0.1	0<br>.0103	$Bi_6O_4(OH)_4(NO_3)$<br>$6H_2O$: Bi-O(1.1)		2<br>.13620	2<br>.27488	0<br>.13868	0<br>.01247
			$Bi_6O_4(OH)_4(NO_3)$<br>$6H_2O$: Bi-O(5.1)		2<br>.35220	2<br>.49088	0<br>.13868	0<br>.01247
			$(NH_4)_{16}$<br>$[Bi_2P_2Mo_{22}O_{39}]_2$<br>$12H_2O$:<br>Bi…Mo(3.1)		3<br>.48980	3<br>.51952	0<br>.02973	0<br>.01806
			$(NH_4)_{16}$<br>$[Bi_2P_2Mo_{22}O_{39}]_2$<br>$12H_2O$:<br>Bi…Mo(1.1)		3<br>.52460	3<br>.55433	0<br>.02973	0<br>.01806
			$(NH_4)_{16}$<br>$[Bi_2P_2Mo_{22}O_{39}]_2$<br>$12H_2O$:<br>Bi…Mo(10.1)		3<br>.87740	3<br>.90712	0<br>.02973	0<br>.01806
			$(NH_4)_{16}$<br>$[Bi_2P_2Mo_{22}O_{39}]_2$<br>$12H_2O$:<br>Bi…O(28.1)		3<br>.90760	3<br>.93733	0<br>.02973	0<br>.01806
$(NH_4)_{16}$ $[Bi_2P_2Mo_{22}O_{39}]_2$ $12H_2O$ (pure final substance)	42.1	0<br>.0187	$(NH_4)_{16}$<br>$[Bi_2P_2Mo_{22}O_{39}]_2$<br>$12H_2O$:<br>Bi…O(10.1)		2<br>.35840	2<br>.33857	-<br>0.01983	0<br>.01783
			$(NH_4)_{16}$<br>$[Bi_2P_2Mo_{22}O_{39}]_2$<br>$12H_2O$:<br>Bi…O(3.1)		2<br>.38810	2<br>.36827	-<br>0.01983	0<br>.01783
			$(NH_4)_{16}$<br>$[Bi_2P_2Mo_{22}O_{39}]_2$<br>$12H_2O$:<br>Bi…O(2.1)		2<br>.44960	2<br>.42977	-<br>0.01983	0<br>.01783
			$(NH_4)_{16}$<br>$[Bi_2P_2Mo_{22}O_{39}]_2$<br>$12H_2O$:<br>Bi…Mo(1.1)		3<br>.52460	3<br>.53179	0<br>.00719	0<br>.01306
			$(NH_4)_{16}$<br>$[Bi_2P_2Mo_{22}O_{39}]_2$		3<br>.48980	3<br>.49707	0<br>.00728	0<br>.01306

Sample	ed. Chi^2	R<br>-factor	Model:<br>Path	N	R<br>$_{eff}$ (model), Å	R<br>$_{fit}$ (calcu-lated), Å	D<br>R, Å	R<br>MSE, Å
			$12H_2O$:<br>Bi…Mo(3.1)					
			$(NH_4)_{16}$<br>$[Bi_2P_2Mo_{22}O_{39}]_2$<br>$12H_2O$:<br>Bi…Mo(10.1)		3<br>.87740	3<br>.88467	0<br>.00728	0<br>.01306

2.3 **SAXS/WAXS curves different periods**

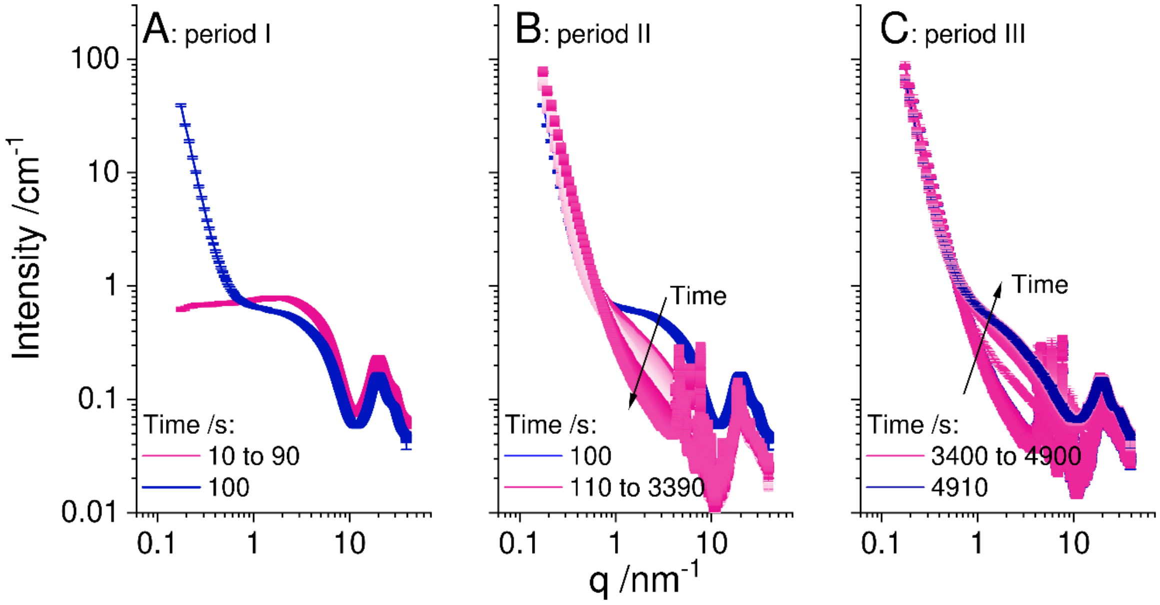

Figure S6. SAXS/WAXS data at different stages of the experiment.

2.4 **MALDI TOF data**

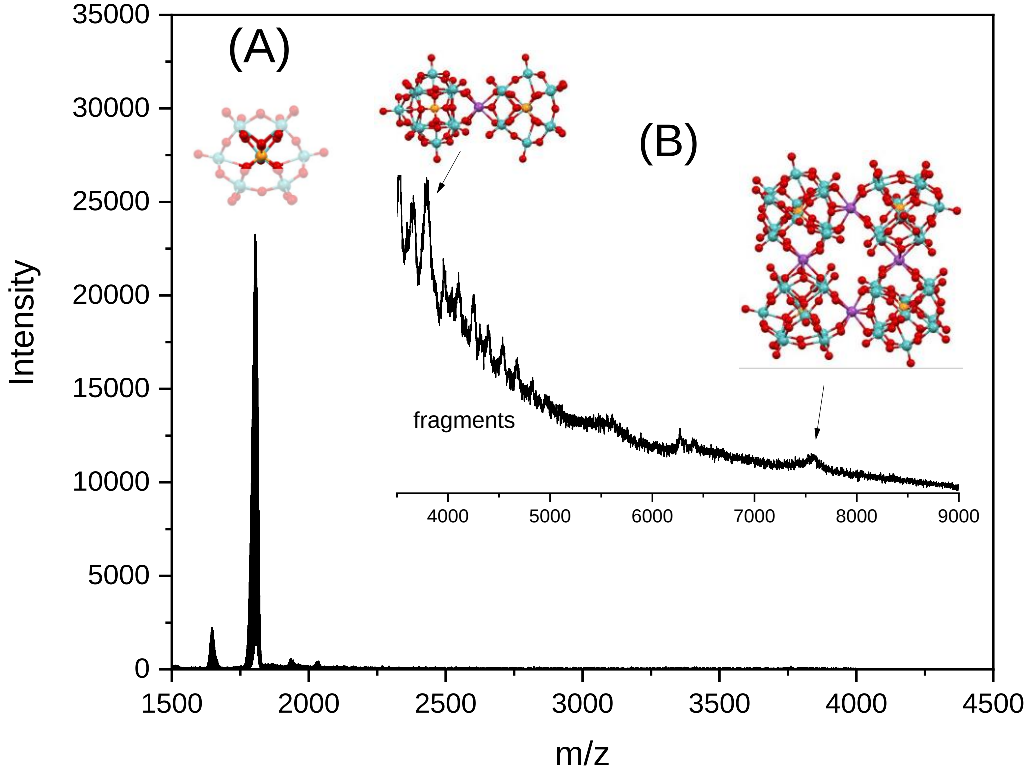

Figure S7. (A) MALDI-TOF mass spectrum of **($P_4Bi_4Mo_{44}$)** and (B) of the final compound (expansion of the *m/z* 3 500-9 000 region).

2.5 TG-DSC-MS, ATR-IR analysis, and temperature-depended XRD

The TG curve of **($P_4Bi_4Mo_{44}$)** recorded in air (Figure S8) exhibits three steps of weight loss. While in the first step (25°C - 220°C) only H_2O is released accompanied by an endothermic effect, N_2O, NO_2 (m/z=46, not shown), and NH_3 are released in the second (220°C - 350°C) and N_2O, NO_2 in the third step (350°C – 485°C) besides H_2O. The formation of N_2O and NO_2 is caused by the oxidation of released NH_3, and is accompanied by exothermic effects at 311°C and particularly by a strong one at 487°C. The nature of the exothermic effect at 311°C could be elucidated by characterization of the decomposition products obtained after the respective TG steps at 220°C and 350°C by means of ATR-IR spectroscopy (Figure S9) and XRD (Figure S10).

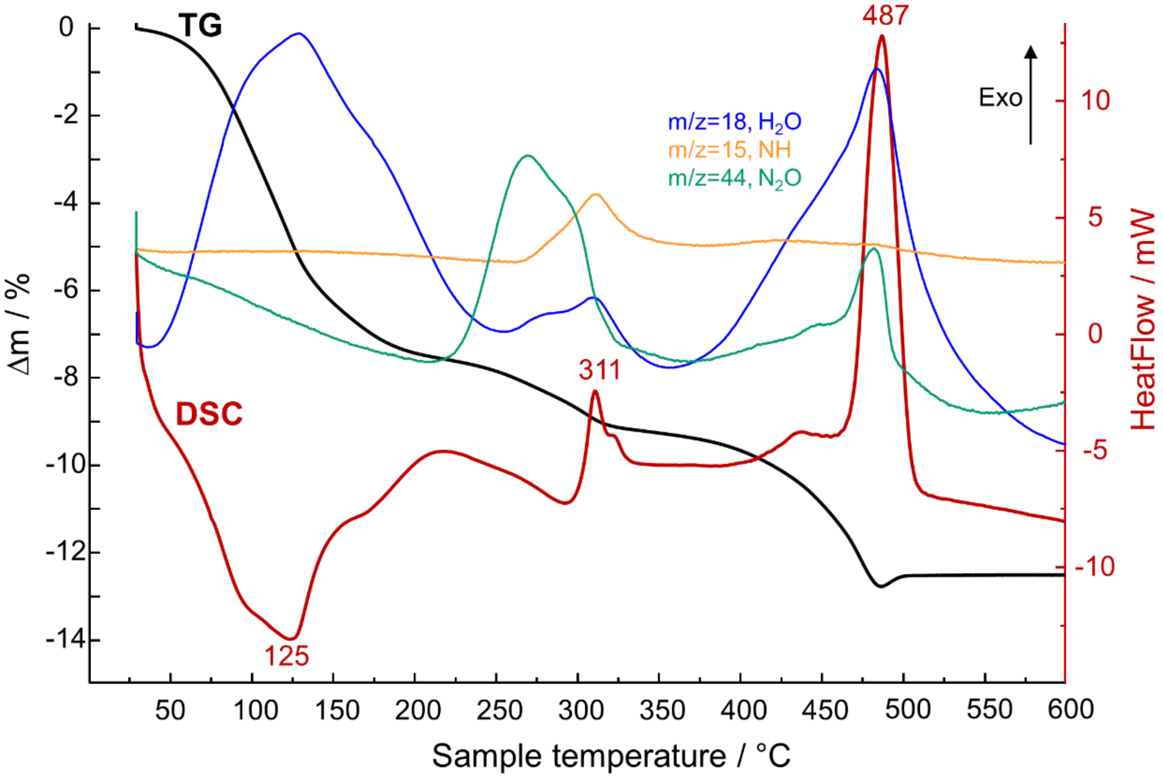

Figure S8. TG-DSC-MS analysis of $(NH_4)_{16}[(BiPMo_{11}O_{39})_4]$ $22H_2O$ **($P_4Bi_4Mo_{44}$)**. Heating rate: 5 K/min, atmosphere: synthetic air (20 ml/min).

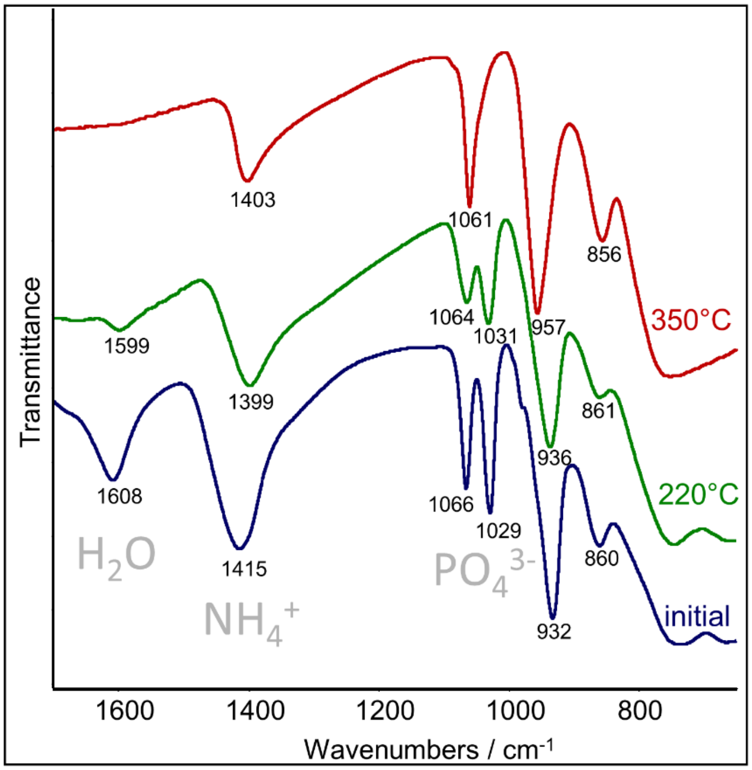

Figure S9. ATR-IR spectra of the initial (**$P_4Bi_4Mo_{44}$**) compound and the decomposition products obtained at 220°C and 350°C, before and after the exothermic effect at 311°C (*cf.* Figure S8).

The product obtained after heating up to 220°C has a similar ATR-IR spectrum as measured for the initial (**$P_4Bi_4Mo_{44}$**) compound showing the typical splitting of the ν(P–O) band as expected for lacunary Keggin ions (Figure S9). After heating to 350°C, only one ν(P–O) band at 1061 cm^{-1} is detected, normally observed for Keggin anions, $[PM_{12}O_{40}]^{3-}$. The respective XRD pattern points to cubic symmetry (*cf.* Figure S10). Both findings indicate the destruction of the tetrameric lacunary Keggin ions and formation of a crystalline phase containing intact Keggin ions.

Such a $\{PM_{11}O_{39}\} \rightarrow \{PM_{12}O_{40}\}$ transformation was also reported to occur during heating of $H_4PVM_{11}O_{40}$, M = Mo, W and during thermal treatment of several lacunary tetrabutylammonium salts.[13] After the last TG step $BiPO_4$ and MoO_3 as final decomposition products were detected in the XRD pattern.

The temperature-depended X-ray study (Figure S10) shows that in the temperature range between room temperature and 50°C the reflections slightly shifted to higher 2θ values, which can be interpreted as decrease of the lattice parameter due to the loss of water.

Within the first temperature range (between room temperature and 50°C) the reflections slightly shifted to higher 2θ values, which can be interpreted as decrease of the lattice parameter due to the loss of water.

Above 45°C, the structure starts to collapse while losing water as evident from the TG-DTA-MS measurements (see Figure S8). Between 100 and 200°C, almost no diffraction signal was detected which could indicate the occurrence of an amorphous phase. At higher temperatures a re-crystallisation starts with changes in the diffraction pattern related to further dehydration shown by TG--MS. The diffraction pattern could be assigned to the Keggin ion structure $(NH_4)_3PO_4(MoO_3)_{12}$ $4H_2O$ (PDF 09-0412). During the last TG event, starting at ~400°C, MoO_3 crystallises followed by $BiPO_4$ (PDF 15-0766) at 450°C. Both phases are stable up to the end temperature of 545°C and can be observed after cooling down the system.

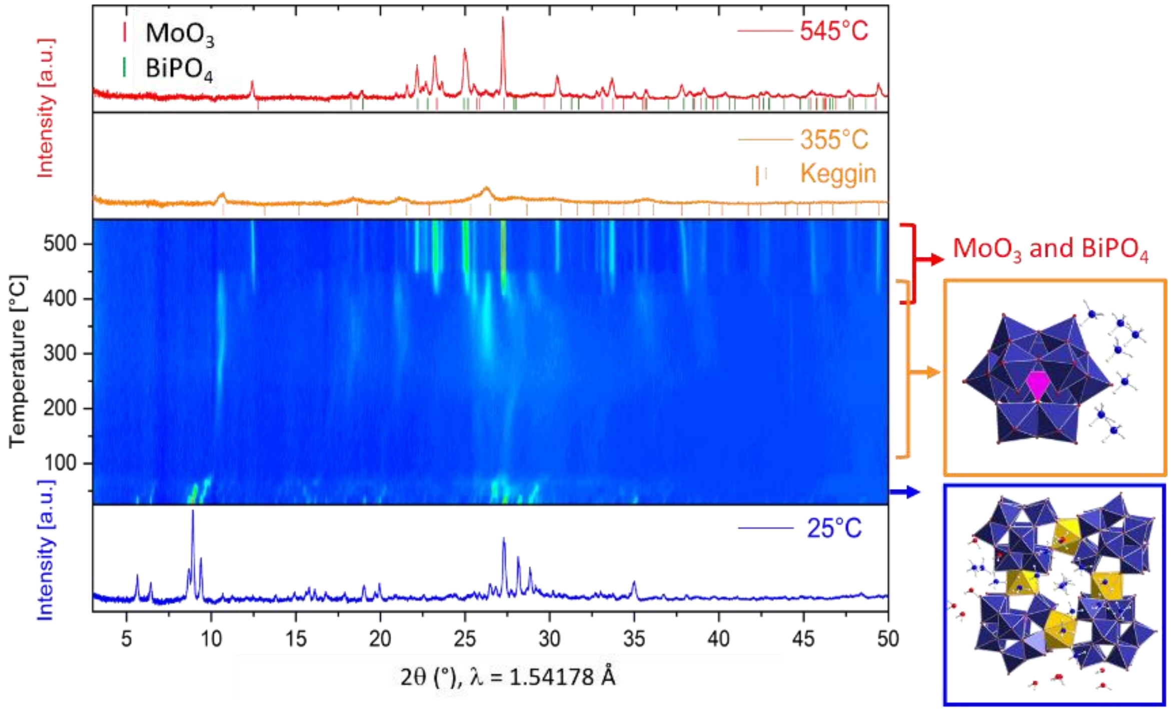

Figure S10. Temperature depended X-ray diffraction patterns recorded during heating of **($P_4Bi_4Mo_{44}$)** in air. The diffraction pattern at room temperature (blue line) is shown at the bottom of the diagram. The formation of the Keggin structure ($(NH_4)_3PO_4(MoO_3)_4$ 4 H_2O) was observed at 80°C persisting until approx. 430°C (yellow line). The diffraction pattern at 545°C (red line) can be assigned to $BiPO_4$ and MoO_4 and is shown at the top of the diagram.

3 References

[1] T. Schmid, P. Dariz, *Heritage* **2019**, *2*, 1662-1683.

[2] T. Schmid, R. Jungnickel, P. Dariz, *J. Raman Spectrosc.* **2019**, *50*, 1154-1168.

[3] (Ed.: B. A. Inc.), Bruker, Madison, Wisconsin, USA, **2016**.

[4] G. M. Sheldrick, *Acta Cryst. A* **2008**, *64*, 112-122.

[5] I. Zizak, *JLSRF* **2016**, *2*, 102.

[6] G. Benecke, C. Li, S. Roth, R. Gehrke, A. Rothkirch, T. Kracht, O. Paris, W. Wagermaier, A. Gourrier, M. Burghammer, in *Emerging Themes in Analysis of Grazing Incidence Small-Angle Scattering Data*, p. 18.
[7] G. Ashiotis, A. Deschildre, Z. Nawaz, J. P. Wright, D. Karkoulis, F. E. Picca, J. Kieffer, *J. Appl. Cryst.* **2015**, *48*, 510-519.
[8] P. H. Eilers, H. F. Boelens, *Leiden University Medical Centre Report* **2005**, *1*, 5.
[9] H. Riesemeier, K. Ecker, W. Görner, B. R. Müller, M. Radtke, M. Krumrey, *X-Ray Spectrom.* **2005**, *34*, 160-163.
[10] M. Newville, *J. Synchrotron Radiat.* **2001**, *8*, 322-324.
[11] N. Ncube, S. Bhattacharya, D. Thiam, J. Goura, A. S. Mougharbel, U. Kortz, *European Journal of Inorganic Chemistry* **2019**, *2019*, 363-366.
[12] M. N. K. Wihadi, A. Hayashi, K. Ichihashi, H. Ota, S. Nishihara, K. Inoue, N. Tsunoji, T. Sano, M. Sadakane, *European Journal of Inorganic Chemistry* **2019**, *2019*, 357-362.
[13] C. Rocchiccioli-Deltcheff, M. Fournier, R. Franck, R. Thouvenot, *Inorganic Chemistry* **1983**, *22*, 207-216; C. Rocchiccioli-Deltcheff, M. Fournier, *J. Chem. Soc. Faraday Trans.* **1991**, *87*, 3913-3920.
